# Survey on Fall Detection and Fall Prevention Using Wearable and External Sensors

**DOI:** 10.3390/s141019806

**Published:** 2014-10-22

**Authors:** Yueng Santiago Delahoz, Miguel Angel Labrador

**Affiliations:** Department of Computer Science and Engineering, University of South Florida, 4202 E Fowler Ave, Tampa, FL 33620, USA; E-Mail: mlabrador@usf.edu

**Keywords:** machine learning, kinect, environment awareness, mobile applications

## Abstract

According to nihseniorhealth.gov (a website for older adults), falling represents a great threat as people get older, and providing mechanisms to detect and prevent falls is critical to improve people's lives. Over 1.6 million U.S. adults are treated for fall-related injuries in emergency rooms every year suffering fractures, loss of independence, and even death. It is clear then, that this problem must be addressed in a prompt manner, and the use of pervasive computing plays a key role to achieve this. Fall detection (FD) and fall prevention (FP) are research areas that have been active for over a decade, and they both strive for improving people's lives through the use of pervasive computing. This paper surveys the state of the art in FD and FP systems, including qualitative comparisons among various studies. It aims to serve as a point of reference for future research on the mentioned systems. A general description of FD and FP systems is provided, including the different types of sensors used in both approaches. Challenges and current solutions are presented and described in great detail. A 3-level taxonomy associated with the risk factors of a fall is proposed. Finally, cutting edge FD and FP systems are thoroughly reviewed and qualitatively compared, in terms of design issues and other parameters.

## 1. Introduction

According to [1], more than 1.6 million U.S. adults are treated for fall-related injuries in emergency rooms each year. The most common consequences of these injuries are fractures, loss of independence, and even death.

Two of the most common causes of falls are due to aging and obstacles in home environment.

As people get older, their bodies go through multiple physical changes making them more fragile, and more prone to falls. For instance, vision deteriorates over time hindering one's capability to explore their surroundings and identify tripping obstacles. In order to minimize the effects of aging as the human physicality decays medications are prescribed. Paradoxically, this increases a person's probability to fall since some drugs reduce mental alertness [1].

A person's living environment is filled with potential fall hazards; which is the reason why most falls happen at home [2]. Slippery floors, clutter, poor lighting, unstable furniture, obstructed ways and pets are common hazards inside a home [3].

References [4–6] suggest conducting a thorough analysis of the house and identifying the possible causes of a fall. From this analysis, a preventative checklist can be created to minimize the risk of a fall. An example checklist might be the following:

- Install handrails on stairs and steps.
- Remove tripping objects (such as papers, books, clothes, and shoes) from stairs and walkways.
- Put grab bars inside and next to the tub or shower, and next to your toilet.
- Improve lighting.
- Wear sturdy shoes both inside and outside the house.
- Train pets. Dogs are more likely to cause a fall than cats.

There exist other ways to prevent fall-related accidents besides home modifications. Motivating patients to exercise, being aware of medication side effects and taking care of vision problems are common methods to avert a fall. Teaching adults how to prevent risk factors is crucial to avoid future falls, since most of them believe that falls are part of aging and nothing can be done about it [7].

Falls have unique patterns and characteristics that can be exploited to detect and predict them. For example, the falling speed rises proportionally with the inertial characteristics of the falling subject. The acceleration is biased to its negative component along the axis perpendicular to the ground. There is also a change in the direction after a fall followed by a period of the subject's inactivity [8].

Fall detection (FD) and fall prevention (FP) systems have been studied for over a decade, the former the most investigated. They share many things in common, for example, they both employ sensing devices to accomplish their tasks. Additionally, they make sense of the collected data through computer vision, data mining and machine learning techniques.

These systems have to overcome many challenges in order to design and implement an effective FD or FP system. Some of the issues they face are the following: obtrusiveness, occlusion, multiple people in the scene (camera-based system), aging (kinematics characteristics change over time), privacy, computational complexity, cost, noise, defining a threshold (threshold-based systems) [9–11]. These and other design issues will be covered in detail in Section 3.

Fall detection and prevention systems have been designed using either external sensors or wearable sensors. External sensors are deployed in the vicinity of the subject of interest (SOI), and wearable sensors are attached to the SOI [12]. There have been also other approaches that use a combination of both types of sensors, known as hybrid systems. This type of systems will not be covered in this review.

Camera-based sensors are perhaps the most common types of sensors used in external sensing. One or multiple cameras are placed in predefined fixed locations where the SOI will perform his/her daily activity. The main drawback of these sensors is their inability to track the user out of the cameras' range of visibility. Another important fact about external sensing is its high cost, as multiple sensors must be purchased to increase the system's coverage [12].

Proximity sensors are another typical example of the external sensors used in fall detection systems. Many of these sensors are usually attached to a walking-aid device, such as a cane or a walker. A fall is detected by measuring sudden changes in the SOI's movements and his/her distance from the proximity sensors. One of the problems these sensors have is their short proximity range; if a person steps away from the walker, the SOI will be out of the sensors' range, which can be misinterpreted as a fall. Also, some proximity sensors can be significantly expensive [13].

Wearable sensors are an alternative to external sensing. They are frequently employed in FD and FP systems. Wearable sensors are attached to the SOI's body, eliminating the space limitation imposed by external sensing. In addition, wearable sensors are generally cheaper than external sensors. The main disadvantage of wearable sensors is their high level of obtrusiveness [12].

Accelerometers are a type of wearable sensors that are widely used in fall detection systems. They are cheap and can be worn on different parts of the body. They are usually embedded in other devices such as watches, shoes, belts, *etc.* With this single sensor most of the SOI's movement characteristics can be extracted and used to detect falls.

Fall prevention systems also take advantage of external sensors and wearable sensors. Similarly, different motion characteristics are extracted from the collected data, which are used to estimate the likelihood of a fall and alert the user in real time.

This paper surveys the state of the art in FD and FP systems, including qualitative comparisons among various studies. It aims to serve as a point of reference for biomedical engineers or anyone interested in conducting investigations on fall-related systems. Section 2 looks closely to the model used by FD and FP systems. Section 3 is dedicated to describe the most important aspects to consider in the design of these systems. Section 4 presents FD systems in detail. Section 5 introduces FP systems. Section 6 presents a qualitative evaluation of FP and FD systems. Finally, Section 7 concludes the paper.

## 2. Machine Learning General Model of FD and FP Systems

**Figure 1.**
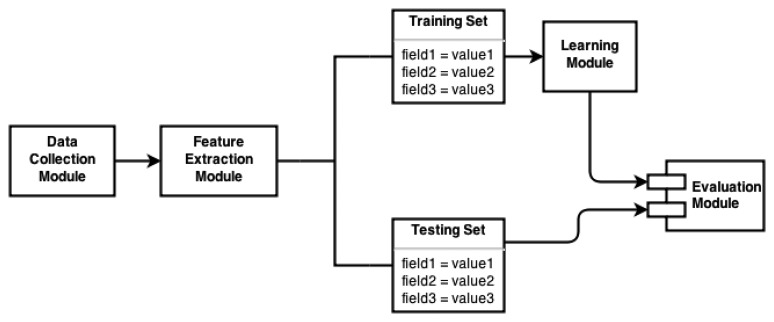
General model of fall detection (FD) and fall prevention (FP) systems.

FD and FP systems use a well defined model to describe the different steps that must be taken in order to detect or prevent falls. Figure 1 depicts the general model employed by these systems. The data collection module is in charge of gathering the SOI's motion data. The feature extraction module selects relevant and more meaningful characteristics that are fed into the next module. Before the learning module is used, the data are split into sets known as the training set and the testing set. Learning algorithms are used to find relationships from the extracted features in the training set. As a result of this process, a descriptive model of the training set is generated. The evaluation model assesses the performance of the generated model by testing its performance with the testing set as input [14]. The following sections will expand on each of the modules.

Before delving into further details about the general model used by FD and FP systems, a brief explanation of key terms is important:

- *Dataset*. The input of any learning scheme. An assembly of similar sets of information. In the context of machine learning, a table formed by *features*, also known as *attributes*, and *instances*. Table 1 shows an example of a weather dataset. It is used to determine if a particular soccer team should play a match or not.
- *Features*. The variables of interest. Outlook, temperature, humidity, *etc.*, in Table 1.
- *Instances*. The determinant of different collection times for the same or different SOI. Rows in Table 1.
- *Class*. Something that needs to be learned or predicted. In Table 1, the class is the attribute “play”, which is used to determine whether the team should play or not.
- *Classifier*. A descriptive model used to predict relationships among the attributes and the class.
- *Feature Space*. Set of all possible instances. Instances are represented as a vector of features < *x*_1_, *x*_2_, *x*_3_, …, *x_n_* >. Each feature can be thought of as a “dimension” of the problem and each example is a point in an *n*-dimensional feature space. For example, the first instance in Table 1 can be represented with the vector of features < *Sunny*,*Hot*, *High*, *False*, *No* >.

### 2.1. Data Collection

The data collection module is in charge of recording specific variables in a systematic fashion. A formal procedure must be used to ensure the accuracy and validity of the collected data.

The whole process starts by identifying the variables needed. In the fall detection context the following variables are commonly used: falling speed, acceleration coordinates, angular velocity, inactivity periods, *etc.* The next step is to determine where and how this data can be gathered. Data can be compiled in different ways such as observing changes, measuring items or weighing items. Noise (meaningless information) is removed from the data; only useful values are used to form the final output data, which must follow a specific format [15,16].

### 2.2. Feature Extraction

Feature extraction is the process by which relevant characteristics or attributes are identified from the collected data [17]. In fall-related systems the acceleration magnitude or the angular magnitude are frequently extracted. Features have to be cautiously picked in order to get a more descriptive and usually smaller output dataset.

The number of features in a dataset has a direct impact on the dataset's descriptive power. The more features a dataset has, the more expressive it is. However, finding meaningful relationships among the instances and the class can be more difficult because the feature space grows exponentially with the number of features.

Feature extraction is also known as dimensionality reduction. Raw data are usually filled with meaningless information. By selecting only the features that best describe the input data and discarding redundant features, the size of the dataset is reduced. This reduction usually lowers the work of the subsequent modules [18].

Although the size of the dataset is usually shrunk (space embedding procedures), there are some methods that enlarge it (non-linear expansions, feature discretization) or even leave it unchanged (e.g., signal enhancement, normalization, standardization) [17].

Feature extraction is commonly divided into two categories: feature construction and feature selection. They are described next.

#### 2.2.1. Feature Construction

The descriptive power of the feature space is determined by how well the collected data are represented. Feature construction is a key element in the data analysis process, and has a significant impact on subsequent stages of the learning endeavor [17].

Realizing an optimal way to represent the input data is domain specific. Most of the time, human experts help in the construction of the feature space. Through their knowledge raw data are converted into meaningful features. Also, their expertise is used to facilitate the task of choosing the adequate variables to observe and not the ones that are irrelevant [19].

Feature construction is also considered as a preprocessing step. The collected data goes through a series of transformations that intend to purify (get rid of irrelevant values) the dataset while preserving the essential information [17]. Some of the preprocessing transformations are the following:

- *Standardization*. Maximize compatibility among attributes. Feature space size does not suffer a change.
- *Normalization*. Attributes are adjusted to share a common measurement scale. Feature space size suffers no change.
- *Signal enhancement*. Used to improve signal-to-noise ratio. Commonly used in images. Normal operations include: baseline or background removal, de-noising, smoothing, or sharpening. The Fourier and wavelet transforms are widely used. Feature space size does not suffer a change.
- *Extraction of local features*. Attributes are encoded with problem specific knowledge. Feature space size can increase or decrease.
- *Linear and non-linear embedding methods*. Used to reduce the size of the original feature space when it is considerably big without losing excessive information. Principal Component Analysis (PCA) and Multidimensional Scaling (MDS) are frequently used.
- *Non-linear expansions*. Increasing the size of the feature space to ameliorate the complexity.
- *Feature discretization*. Continuous data can be troublesome for some algorithms. It helps in the description and understanding of the data. Feature space is enlarged.

#### 2.2.2. Feature Selection

Knowing how pertinent or relevant a feature is, is essential to have a more descriptive feature space. Feature selection is in charge of eliminating redundant and meaningless values without losing significant information [17]. This translates into storage space savings and processing algorithms' speed.

Some of the feature selection strategies used nowadays are briefly introduced next.

- *Filters*. Ranking method. A relevance index is used to assign an order of importance to the attributes. Use of classical statistical tests: T-test, F-test, Chi-squared, etc.
- *Wrappers*. Predictor or classifier is involved in the selection process. Subsets of features are chosen and a learning scheme is evaluated with them. Scores are assigned to the features depending on the accuracy of the predictor.
- *Embedded methods*. Predictor or classifier is involved in the selection process. Similar to wrappers. They are specific to given learning machines.

### 2.3. Learning Module

A machine in this context is an automated system that may be implemented through software. Machine learning focuses on finding relationships in data and analyzing the processes to extract such relationships.

There are two types of learning mechanisms, namely supervised and unsupervised. The former creates a prediction scheme using labeled data. The dataset is usually split and one part is used for training and the rest for testing [20]. The latter creates a recognition model using unlabeled data. Every activity is weighted based on a probability that is assigned manually. It uses a pre-established model and the current state of the system to update these weights every time a new observation is detected [20].

While supervised learning is widely used in fall-related event systems, hardly any information can be found about unsupervised learning. Unsupervised learning will not be covered in this review.

In the next section, the most relevant supervised learning algorithms are described.

#### 2.3.1. Decision Trees

Decision trees (DTs) are one of the oldest algorithms used in supervised learning. These algorithms use a *divide and conquer* approach, and their basic data structure is a tree that most of the time is binary [18].

The C4.5 algorithm, created by Ross Quinlan generates a decision tree that can be used to solve classification problems. The construction of the tree is performed in a recursive manner [21]. An attribute is chosen as the root of the decision tree, and branches are created for every value of the attribute. The process is repeated on every branch using the remaining attributes that reach them. The stopping criteria is met when all of the instances in the branch have the same class or there are no more attributes left [18].

The selection of the nodes (attributes) is determined using a measurement of the purity of all possible nodes. The level of purity associated to each node is just the number of instances in the node that have the same class. The more instances of a given class the node has, the more pure this will be. C4.5 uses *information gain* to measure the purity of the nodes [18]. Algorithm 1 shows the steps to calculate the information gain of a node.



**ALGORITHM 1:** Information gain pseudocode.
 **Input:** Dataset D= { (*x*_1_,*c*_1_),…,(*x_N_*,*c_N_*) } *Output:* predicted class C_i_. **for**
*each attribute or node i*
**do**  Calculate the information of each branch  Average information of all attribute values or branches, *avg*  Calculate the information of the unsplit node, *unsplitINFO*  Calculate the *information gain of the node i*:   Gain[node] = *unsplitINFO* — *avg* **end** **return** Gain[node]


#### 2.3.2. Naive Bayes

**Figure 2.**
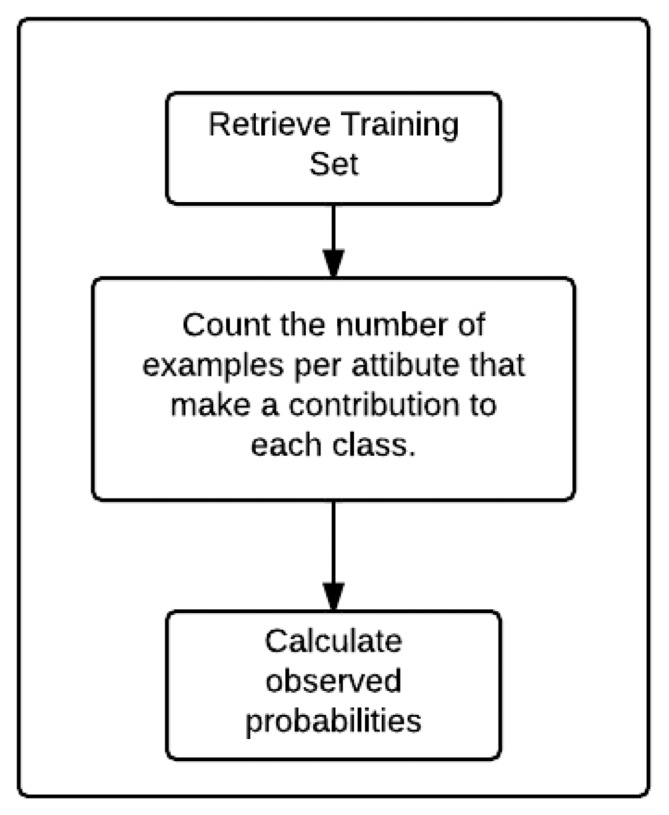
Naive training algorithm block diagram for a binary class dataset [18].

This is a very popular supervised algorithm that uses a probabilistic model based on the Bayes Theorem [18]. It is said to be naive because independence is assumed among the different attributes within the dataset. The common data structures used are matrices, and its algorithmic design approach is usually a straightforward or brute force one. Its time complexity (*O*(*T A*)) depends on the number of training examples (T) and the the number of attributes or features (A). Figure 2 shows a basic block diagram of the learning algorithm.

#### 2.3.3. *K*-Nearest Neighbor

This algorithm belongs to a subgroup of supervised learning algorithms known as instance-based classifiers. New and unseen instances are compared with instances that are stored in the training set.

*K*-nearest neighbor algorithms are also called lazy classifiers because there is no training involved. The basic algorithm uses the closest neighbors of the not yet classified new instances to classify them. Every time that a new example needs to be classified, it is compared with all the examples in the dataset. Consequently, *k*-neighbor algorithms use a straightforward approach to solve classification problems [18].

Suppose there is a dataset with *n* classified examples and the only categorical or nominal attribute is the class, the rest are real value attributes. Each classified example acts as a point in the feature space. A way to calculate the *k*-nearest neighbors for unclassified examples would be to find the *k* already classified examples that are closest to the unclassified data. Once the *k* neighbors have been identified, a majority class vote will take place among them to classify the new instances. Since the attributes are numeric, distance measurements can be used to determine which are the *k* closest neighbors. Euclidean, Manhattan and city-block distances are commonly used in *k*-nearest neighbors algorithms [18].

On *k*-nearest neighbor algorithms, most of the time, the information or collected data are stored in matrices. Moreover, since every instance must be checked in order for a new entry to be classified, the time complexity of these algorithms is equal to *O*(*n*^2^), where *n* is the number of classified examples [18]. A basic *k*-nearest neighbor pseudocode is shown in Algorithm 2.



**ALGORITHM 2:**
*K*-nearest neighbor pseudocode.
 **Input:** Dataset D= { (*x*_1_,*c*_1_),…,(*x_N_*,*c_N_*) }, and unlabeled instance *x*=(*x*_1_,…,*x_N_*). **Output:** predicted class *C_i_*. **for**
*each classified example (x_i_,c_i_)*
**do**  calculate distance d(*x_i_*,*x*)  order d(*x_i_*,*x*) from lowest to highest  select k nearest neighbors to x  vote for majority class among k neighbors, *C_i_*  **return**
*C_i_*.**end**


#### 2.3.4. Support Vector Machines

Support vector machines (SVMs) are a relatively new type of supervised machine learning algorithms, and they outperform many of the classic algorithms. In a two-class classification problem, the main goal is to create a model that places every new example in the correct class. SVMs algorithms try to solve this problem by taking the training examples into a higher dimension where they are linearly separable and can be assigned to a class with little uncertainty [18].

Binary class datasets that are linearly separable are easy to classify because the decision boundary of the two classes is just a straight line or plane that divides the feature space into two regions: class A and B.

In SVMs, the input space is transformed into a higher dimensional space using a non-linear mapping. The idea is to take the instances from the original feature space where they are not linearly separable to a new feature space where they are. On this new space, a hyperplane (a straight line in 2 dimensions) is created, and it works as a decision boundary that separates the data; this boundary is also known as the *maximum margin hyperplane*.

**Figure 3.**
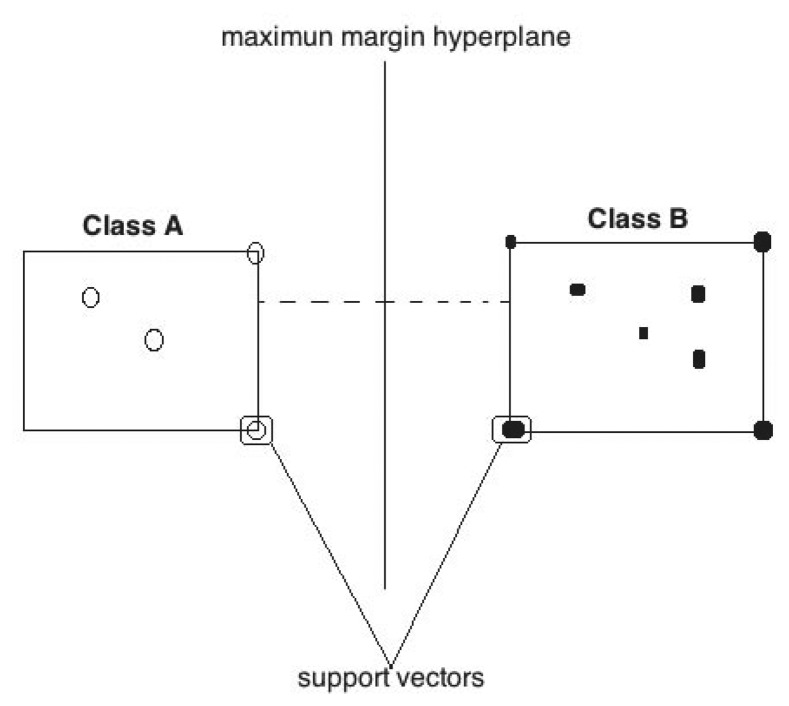
SVMs maximum margin hyperplane [18].

The training points that are closest to the decision boundary are called *support vectors*. The support vectors uniquely define the maximum-margin hyperplane for the learning problem. In this manner, support vector machines search for a maximum margin hyperplane to separate the data with the examples on the border called support vectors. Every new entry will be taken to this new space where it will be classified depending on the region it falls into [18]. Figure 3 shows an example of a decision boundary.



**ALGORITHM 3:** SVMs training pseudocode.
 **Input:** Training data **Output:**
*Maximum margin hyperplane*, *H_max_*. calculate support vectors *a*(*i*) calculate maximum-margin hyperplane, *H_max_* **return**
*H_max_*


The math involved in SVMs is extremely complex and therefore difficult to implement [22]. The steps in order to realize a SVM training algorithm are described in Algorithm 3.

Calculating the *maximal margin hyperplane* can be achieved by solving Equation (1).

(1) $$\begin{array}{c} \;x=b+\underset{\text{i is support vector}}{\text{∑}}\alpha_{i}c_{i}a(i)\cdot a\\ \text{where}\\ \begin{array}{ll} b & =\text{Numeric parameter}\\ \alpha_{i} & =\text{Numeric parameter}\\ \alpha (i) & =\text{Support Vector}\\ a & =\text{Test vector} \end{array} \end{array}$$

Finding *b*, α*_i_* and the support vectors *a(i)* is a type of optimization problem known as *constrained quadratic optimization*. There are currently two approaches to solving the quadratic optimization problem: the primal form and the dual form. The former transforms the original equation into a form that can be solved by quadratic optimization methods. The latter rewrites the classification rule into its unconstrained dual form, where the problem depends only on the support vectors [22,23].

In [23], an analysis of both kinds of implementation is given. They concluded that for a dataset or matrix of *a* attibutes (columns) and *n* examples (rows), the training computational time complexity is equal to *O(na^2^* + *a*^3^) for the primal form and *O(an^2^* + *n^3^)* for the dual form.

Table 2 compares the most relevant characteristics of the supervised learning algorithms reviewed.

### 2.4. Model Evaluation

Evaluation is a fundamental module in the learning process because it permits to systematically assess different learning techniques and compare them with one another [18].

Performance indicators are frequently used to measure the efficiency of the inferred structure [24]. The error rate is a common predictor employed in the assessment of learning machines. The system predicts the class of each instance: if it is correctly classified is marked as a *success*, otherwise is marked as an *error*. So, the error rate is a ratio of the errors over the whole set of instances.

As stated before, the dataset is usually split and one part is used for training and the rest for testing. The latter is a considerable conditioning factor in the performance of the system. For instance, using the training set for testing purposes can be misleading; the estimated performance is highly optimistic.

Another way to measure the performance of the system is through cross-validation. A fixed numbers *of folds* or partitions of the data are selected, some are used for training and the rest for testing. The process is repeated many times until all of the folds (group of instances) are used exactly once for testing. The results of every iteration are averaged and an overall error rate is calculated.

Cross-validation is perhaps the method of choice in most of fall-related systems. Statistical tests are usually used with cross-validation to compare the performance of a classifier for a particular dataset [12].

**Figure 4.**
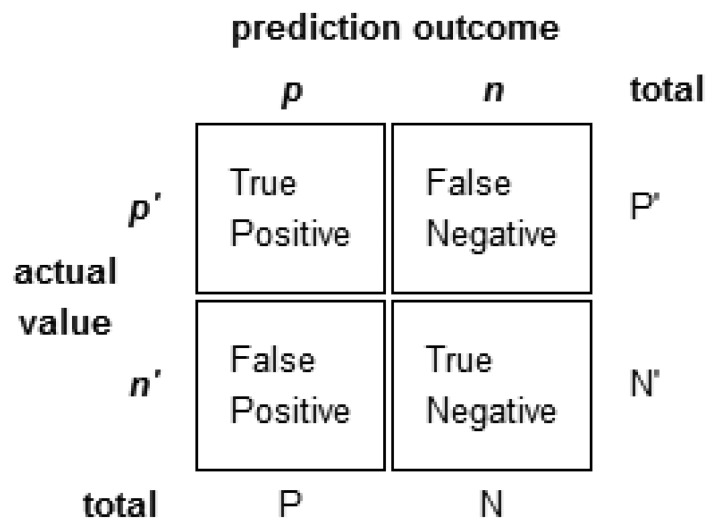
Confusion matrix example.

The results of a classifier are commonly stored in an array known as *confusion matrix*. It allows to visualize the learning algorithm's performance in a specific table. An example of a confusion matrix is depicted in Figure 4.

- *True Positives* (TP): Number of positive instances that were classified as positive.
- *True Negatives* (TN): Number of negative instances that were classified as negative.
- *False Positives* (FP): Number of negative instances that were classified as positive.
- *False Negatives* (FN): Number of positive instances that were classified as negative.

Performance indicators used to evaluate the efficiency of learning algorithms are presented next.

The *accuracy* of the system is the most extensively used performance indicator in classification problems. It is defined as follows:

(2) $$Accuracy=\frac{TN+TP}{TP+TN+FP+FN}$$

The *recall* or *sensitivity* or *true positive rate* is the ratio of the correctly classified positive instances over the entire set of positive instances.

(3) $$\text{Recall}=\frac{TP}{TP+FN}$$

The *precision* or *positive predicted value* is the ratio of the number of correctly classified positive instances to the entire set of instances classified as positives.

(4) $$\text{Precision}=\frac{TP}{TP+FP}$$

The *specificity* (SPC) or true negative rate is the proportion of the correctly classified instances as negative over the entire set of negative instances.

(5) $$SPC=\frac{TN}{FP+TN}$$

The *fall-out* or *false positive rate* (FPR) is the proportion of the incorrectly classified instances as positive over the entire set of negative instances.

(6) $$FPR=\frac{FP}{TN+FP}$$

A way to combine this indicators is through the *F-measure* (a measure of a test's accuracy).

(7) $$F-measure=2\cdot \frac{Precision\cdot \text{Recall}}{Precision+\text{Recall}}$$

**Figure 5.**
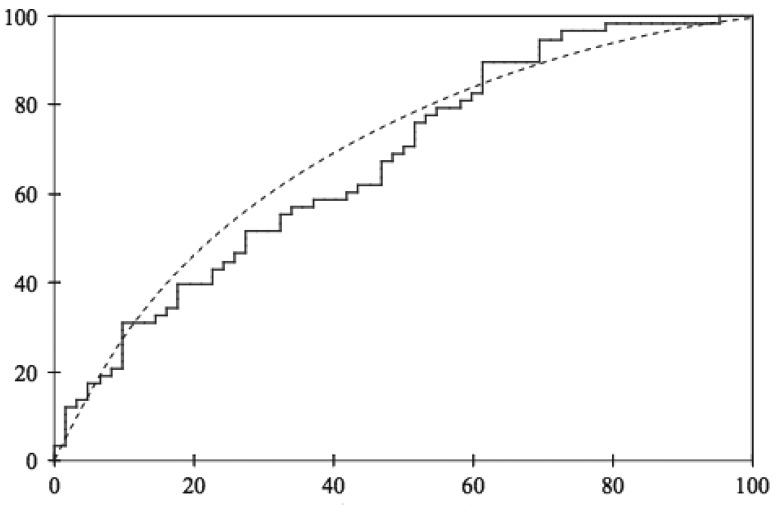
ROC curve example.

ROC (Receiver Operating Characteristic) curves are also used as a tool for diagnostic test evaluation. The performance of a binary classifier is plotted as the classifier's discrimination threshold is varied. It is created by plotting the recall vs. the fall-out, at various threshold settings. The threshold is used to determine the class the current instance belongs to, when the output of the classifier is a real value (continuous output). Figure 5 shows an example of a ROC curve.

## 3. Design Issues

In this section the most important aspects that need to be considered when designing or evaluating FD and FP systems are described.

### 3.1. Obtrusiveness

People tend to use things that are comfortable and non-invasive. Neither fall detection nor fall prevention systems should require people to wear or to interact with devices. This is even more important on monitoring systems, where the subject is under surveillance 24/7. Camera-based systems do not suffer from these types of problems since no object is attached to the user. Wearable sensors have to deal with this problem though. The level of obtrusiveness varies from system to system and in the number of sensors used. While some systems only require the user to wear simple items like a wrist band or a belt, others may require additional gadgets.

There is no doubt that embedding the analysis and classification modules in the data collection module results in a more robust and responsive system, since it will not depend on unreliable wireless communications that might be unavailable or prone to errors. However, this usually means bigger and more obtrusive devices.

### 3.2. Occlusion

Line of sight obstructions are a major problem in camera-based systems. If the subject is being blocked by an object, no analysis of his/her current activity can be performed. In fact, the entire system becomes useless at that point. The work in [9] suffers from occlusion since subjects can sometimes be behind a sofa or furniture while been monitored. The most common solution is to place multiple cameras in different areas of interest to widen the visibility range so the target is visible from any angle. The drawback of this solution is the increase in computing complexity and cost.

Depth cameras are used to mitigate occlusion problems. These cameras make use of two or more sensors to generate 3D depth perception. Images are created using range imaging techniques. Some of these techniques are [25]: stereo triangulation, sheet of light triangulation, structured light, and time of flight. Stereo triangulation is achieved by finding the corresponding points in two frames from two different cameras. In sheet of light triangulation, a source of light is projected into the scene creating a reflected line as seen from the source; the shape of the reflected line is used to measure the distance of the light source and the reflected points. Structured light works similar to sheet of light triangulation but the scene is illuminated with a predefined light pattern. Time of flight is similar to a radar, but it uses a light pulse instead of an RF pulse; it measures the distance of the objects in the scene based on the speed of light.

A typical example of a depth camera is the popular Microsoft Kinect. It uses two sensors (an infrared projector and an infrared sensor) to generate a 3D map of the scene [26]. The use of a 3D map allows to differentiate between obstructions and the subject (depending on the camera position); therefore, further analysis can be done on the current activity of the subject.

### 3.3. Multiple People in the Scene

Systems that utilize cameras to track a person usually assume that only the targeted subject will be captured. In normal life scenarios, multiple people live in the same place and share the same environment. If there are multiple people in the scene the system might suffer from occlusion, because one person might be partially blocking the line of sight to another person. A possible solution is to use additional cameras placed in different locations to get a different perspective of the same scene.

### 3.4. Aging

Fall-related systems depend on people's movement patterns to track their fall susceptibility. However, human body deterioration leads to changes in the kinematic characteristics of a person over his/her life span. For instance, if a person's gait changes, his/her previous gait data lose their value since that information can no longer be used as “ground truth” to draw conclusions about his/her walking behavior. When these changes are detected, a new set of data has to be collected and used as a reference to further analyze the kinematic behavior of the target.

### 3.5. Privacy

One of the main problems of camera-based systems is the fact that people do not like to be watched; they are worried that their intimacy will be violated. Few studies address this issue. The most common way researchers approach this issue is by only sending subjects' images when an alarm is triggered. Ozcan *et al.* [10] developed a fall detection system that utilizes a wearable embedded smart camera that is mounted on the waist of the subject of interest. The camera captures the images of the environment and not the subject's, eliminating the privacy concern. Gasparrini *et al.* [26] developed a fall detection system using a depth camera (Microsoft Kinect). Privacy issues are not a problem due to the fact that this cameras do not recognize the facial characteristics of the subject.

### 3.6. Computational Cost

It is crucial to determine if activity classification will be performed on a server or in the data collection device. A server is usually an entity with huge processing power, storage and energy capabilities that are suitable for performing complex computations. On the other hand, data collection devices are restricted in their processing power, storage and energy consumption and are usually not suitable to perform complex operations. For instance, classification algorithms such as Instance Based Learning and Bagging [18] are very computationally intensive in their evaluation phase, which makes them not suitable for use in data collection devices.

### 3.7. Energy Consumption

Communicating the kinematic information of a subject over a wireless network consumes a lot of power on mobile devices, so energy must be carefully scheduled to minimize its use. Extending the battery life can sometimes be advantageous if the size and weight of the device does not present a problem.

Some of the factors that have a direct impact in the energy consumption of a fall-related system device are: Communication type, number of sensors or cameras, number of images.

Communication is usually the most energy consuming operation, therefore it is necessary to create communication strategies to minimize the amount of transmitted data. Short range wireless technologies (Bluetooth, Wi-Fi, *etc.*) should be chosen over long range technologies (Cellular, WiMAX, *etc.*) since they use less power. There exist other methods to reduce the energy consumption but jeopardize the system's performance, like data aggregation and compression. Another way to save energy is by performing the analysis and classification stage in the data collection device, so no data has to be sent to a server [27].

The number of sensors or cameras used have a direct impact on the energy consumption of a system. It is obvious that the more sensors or cameras a system has the more energy energy it consumes. There are occasions where not all sensors or cameras are necessary and they can be turned off contributing to energy savings.

The frame rate and image resolution are directly related with the amount of energy consumed by a system. The more pixels are processed the more energy the system utilizes. So compacting images and choosing an appropriate frame rate is crucial to decrease the energy used.

### 3.8. Noise

Noise is considered as all external signals that distort the signals being monitored. There are different types of noise depending of its source but in general they all affect the proper observation of variables of interest. The most common sources of noise in wearable sensors (e.g., accelerometers) are mechanical and electrical thermal noise. The former is related to the mass and mechanical resistance of the sensor's seismic system. The latter is related to internal or external electronics used in the measurement device [28].

The following are noise considerations when using accelerometers that are worth mentioning. Cables should be as small as possible to minimize the effect of the cable's capacitance noise. Devices should be shielded to reduce the noise generated by external signals. The selection of accelerometers should be based on their noise specifications [28].

Noise can also be found in images as random brightness variations or color information that generates undesirable results. In [9], a computer-vision fall detection system, image noise is eliminated by removing blobs with a size smaller than 50 pixels.

### 3.9. Defining A Threshold

Threshold-based systems use thresholds to draw conclusions of an event; setting these thresholds can be a considerable challenge. Thresholds depend on the method used, fall type (fall forward, fall backward, *etc.*), and the physical characteristics of the subject being monitored. The rules applied to a tall person are not usually the same for a short one and viceversa. The common approach is to set a threshold that generalizes as much as possible a targeted population.

## 4. Fall Detection Systems

**Figure 6.**
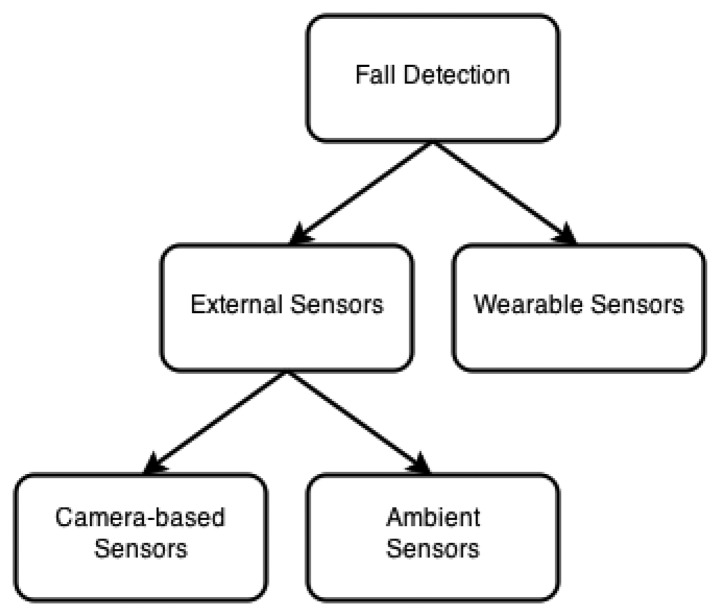
FD sensors.

The following sections present the basic structure of a FD system and the different types of sensors (external or wearable) that are used. Figure 6 [14] shows a flow chart of the sensors that will be discussed here.

### 4.1. External Sensors

External sensors are devices that sense information about the environment and are commonly used to capture the SOI's movements in order to detect falls. There exist two types of external sensors, namely camera-based sensors and ambient sensors. Both types of sensors will be covered next.

#### 4.1.1. Camera-Based Sensing

Fall accidents happen in fractions of seconds, typically between 0.45 and 0.85 s [29]. During the incident, the posture and shape of the faller change drastically. These sudden changes are key to determine if a fall occurred. Camera-based systems benefit from these patterns by monitoring the subject's posture and shape during and after a fall.

Dealing with images consumes a considerable amount of processing power. The frame rate (images per second) of a video is usually 24 frames per second (FPS); on each frame, camera-based algorithms process images pixel by pixel. A common digital image can have 720 × 480 pixels, that is 345,600 pixels that must be analyzed in 41.6 milliseconds, which requires a great deal of computational power. To mitigate this computational cost, images are usually compacted and redundant pixels are discarded in a pre-processing stage.

Camera-based fall detection systems use different approaches to detect falls. For example, some systems such as the ones described in [29,30] are based on human skeleton. They are robust solutions but their computational cost is unattractive for real time applications. Others systems are based on simpler features (e.g., the falling angle, vertical projection histogram) [31,32]. They are not as computationally intensive as the human skeleton based systems but they suffer from a high false alarm rate.

Privacy issues are one of the biggest concerns for camera-based systems. Few solutions to alleviate this problem are known. In [10], Ozcan *et al.* developed a fall detection system that utilizes a wearable embedded smart camera that is mounted on the waist of the SOI. The camera does not record the SOI's motion pictures but the environment's around him/her. Other possible solutions use infrared cameras [33] and thermal cameras [34].

#### 4.1.2. Ambient Sensing

The environment surrounding a subject of interest is usually targeted to track his/her falling behavior. Multiple sensors are deployed in the vicinity of the user, and they are used to collect information about the user's interaction with the sensors.

Pressure sensors are frequently used because of their low cost and non-obtrusiveness. The basic principle is that the pressure changes when the user is close to a sensor; the closer the user is to the sensor the higher the pressure is. The main drawback these sensors have is their low fall detection accuracy (below 90%).

Alwan *et al.* [35] developed a floor-vibration fall detector. The system relies on the floor vibrations produced by humans when performing daily activities. It uses the vibration patterns of the floor to detect a fall.

Sixsmith *et al.* ([36,37]) introduced a fall detection system based on Pyroelectric IR sensor arrays. The arrays only track moving warm objects. Two characteristics are analyzed to detect falls: target motion and inactivity periods.

In [38], a bed exit detection apparatus is presented. The device detects the presence of a patient on a patient-carrying surface. It is used to assist caregivers in determining when patients fall and their falling patterns.

### 4.2. Wearable Sensors

Wearable sensors are electronic devices that are worn by a SOI, and they collect data about the SOI's motion characteristics. Besides their small size, these sensors are typically of low cost, making them an attractive solution to low-budget projects [39].

Accelerometers are the most common wearable sensors used nowadays. They are small in size and cheap; they can be easily placed in any part of the human body. The pelvis of a subject is a common location because the center of mass can be easily calculated [40–42]. Other frequent positions are the wrist and the ankle.

Most of the studies in the literature use accelerometer data along with threshold-based algorithms to detect fall-related events. A threshold is a limit that when surpassed triggers an action in the system (e.g., a fall is detected—caregiver is informed). The most common threshold used with the accelerometer data is the sum vector magnitude of acceleration, which describes the activity level of the body, given by Equation (8).

(8) $$a=\sqrt{a_{x}^{2}+a_{y}^{2}+a_{z}^{2}}$$

Chen *et al.* [40] developed a fall detection system using a tri-axial accelerometer. The sensor is mounted on the subject's pelvis, and it is used to identify human movements. Eight fall scenarios were used: stand still, sit to stand, stand to sit, walking, walking backwards, stoop, jump and lie on the bed. The solution proposes a three-level fall detection criteria that includes a sum vector magnitude of acceleration, acceleration on the horizontal plane and the reference velocity. The first one describes the spatial changes of the acceleration while falling. The second one measures the body inclination. The last one is used to determine if the subject is at rest or not. The results of this study show the effectiveness of the accelerometer data to detect falls.

**Figure 7.**
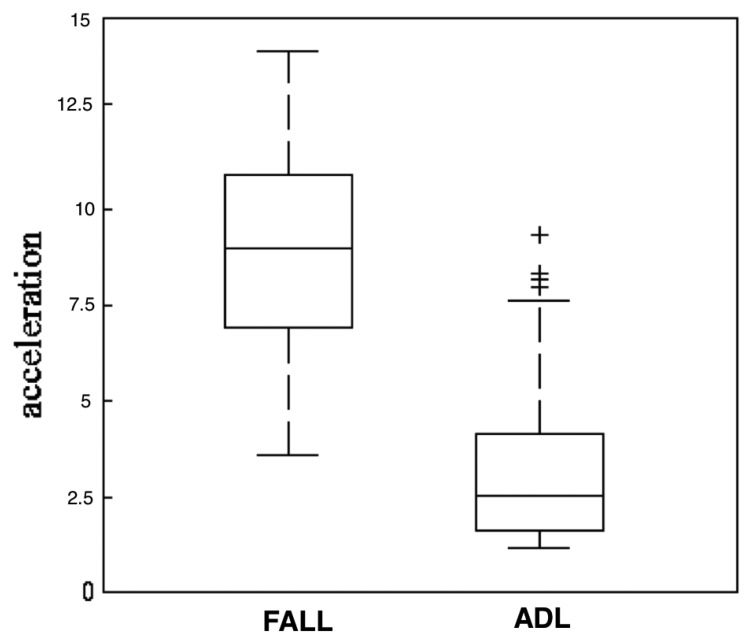
Falls *vs.* normal activities. Acceleration data [42].

While it is true that falls generate higher accelerations than other activities, there are others that have similar accelerations patterns such as jumping, climbing stairs, sitting or standing up quickly. This overlapping data poses a problem when distinguishing a fall from activities of daily living (ADL), and it is the main drawback threshold-based systems have [42]. Figure 7 shows acceleration data collected from 10 subjects during falls and while doing normal activities as explained in [42].

The use of machine learning techniques with acceleration data is an approach that does not necessarily employ a threshold-based solution to detect falls. Vallejo *et al.* [42] proposed an alternative fall detection method that uses artificial neural networks. The system consists of a 3D accelerometer mounted on the waist, a microcontroller, ZigBee module, some LEDs and a lithium battery. The artificial neural network is fed with the data collected, and it was able to classify if the person was performing a normal activity or if a fall occurred. The results of the experiments show that machine learning does not require threshold-based solutions to detect falls.

## 5. Fall Prevention Systems

According to [43], $43.8 billion dollars are estimated to be used in fall-related medical care expenditures by 2020. It is of extreme importance to find ways to minimize these costs, and at the same time, improve the quality of life of the people who suffer the consequences of a fall [43,44]. The easiest way to accomplish this is through prevention.

Educating and discussing the potential consequences of a fall with the patients constitute the principal method doctors and caregivers have in order to prevent falls. Although important, this solution is not enough, more non-patient dependent strategies must be considered.

Care givers have to identify all of the scenarios and circumstances surrounding a fall and provide a framework to prevent them. This framework must be constructed based on data acquired from various scenarios surrounding fall-related events.

Most of fall-related events are usually collected through questionnaires, fall diaries, and/or phone calls. Although these data collection practices do provide relevant information, the information is not always reliable. This happens because people often forget or remember incorrectly the exact conditions of their fall [43].

People do not just fall; there is always an underlying cause or risk factor involved in every fall. The more risk factors there are, the more a person is susceptible to experiencing a fall.

**Figure 8.**
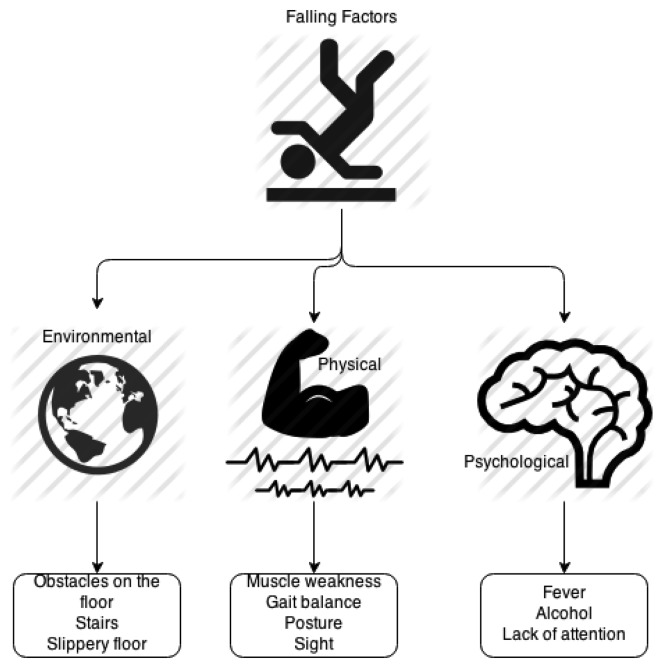
Falling factors.

Falls are roughly associated with three risks factors: environmental, physical, and psychological factors. The first, environmental conditions, are obstacles on the floor, climbing or descending stairways, slippery floors, *etc.* The physical factors are those related to a patient's body condition such as muscle weakness, gait balance, posture, sight, *etc.* Psychological factors deal with things that temporarily affect people's minds such as fever, alcohol, lack of attention, *etc.* Most of the times only psychological and physical falling factors are tackled [45]. Figure 8 depicts the taxonomy for fall prevention systems.

The following sections expand on the aforementioned risk factors.

### 5.1. Environmental Factors

Environmental hazards are obstacles, bumps, or any tangible object that causes a fall. Most older adults come in contact with these hazards in their homes [2]. This is the reason why home safety assessment and household modifications have been proposed as a way to minimize the risk of falls [3].

Home hazards alone do not seem to be enough to cause a fall, but the exposure to environmental risks have a greater role in fall events for the elderly population. A review of the efficacy of home modifications for fall prevention was proposed by [3]. They assessed the contribution of environmental hazards to fall-related accidents and the advantages of environmental interventions to reduce falls. Six case-control studies were reviewed in their assessment. Not surprisingly, they found that frail people have a higher tendency to experience a fall than vigorous ones. However, they claimed that energetic older people are more susceptible to environmental hazards due to their risk-taking behavior. They finished their assessment with the following conclusions:

- “Household environmental hazards may pose the greatest risk for older people with fair balance”.
- “Reducing hazards in the home appears not to be an effective fall-prevention strategy in the general older population and those at low risk of falling”.
- “Home hazard reduction is effective if older people with a history of falls and mobility limitations are targeted”.

A concept introduced in [8], not implemented though, proposes the addition of localization features to a fall prevention system. The idea is to assess the risk of a fall depending on the current location of the subject. There are some areas of a household where the risk of experiencing a fall is higher (e.g., bathroom, stairways) [3]. Whenever the subject is close to one of these areas, he/she is informed about the risk associated with the place. The purpose behind this approach is to increase the subject's awareness and his/her disposition to be careful.

Environmental fall prevention systems strive to gather enough data from the subject's surroundings in order to identify and avoid fall hazards in real time. They also alert the subject of possible tripping obstacles in his/her way.

The environmental fall prevention area of research has been ignored over the years because almost no study can be found in the current literature.

### 5.2. Physical Factors

As people get older, their bodies start to lose the vitality they once had; in addition to this, medical conditions start to arise. Due to lack of exercise and the natural deterioration that comes with age, muscles, bones and other parts of the body weaken; which leads to many problems including falling [6].

Over the last years, intelligent walking-aid devices have been made available to assist patients who are prone to falling in their daily life. They take advantage of robotic advances in motion control to create responses based on human intentions. These walking-aid devices not only offer physical support to the patients, but also prevent falls by detecting possible hazards around the subject [4].

Intelligent walkers have been used during the last decade to assist and/or rehabilitate people experiencing weakness in their lower limbs. These types of walkers offer obstacle avoidance, path following, gravity compensation, and many other features. They fall into two categories: active and passive walkers. The first one uses servo motors and the second one does not. Active walkers are robust, but they can be dangerous if not calibrated properly because they can move unintentionally. Passive walkers, on the order hand, are safer but not as sturdy as the active ones due to their light weight.

RT walker [30] is a passive-type walker which only uses servo brakes. It employs the user's dynamics and movement directions as feedback to realize the different motion characteristics. An estimation method based on the user's state is used to get his/her motion features. Three user's states were utilized; these states are stopped, walking, and emergency. The distance between the user and the RT walker, and the walker's velocity are used to determine the current state. In the stopped and walking state, the risk of falling is at a minimum. In the emergency state, the apparent dynamics of the walker are modified by controlling the brake torques of the wheels, thus preventing the fall. The prototype consists of a support frame, two wheels with powder brakes, two passive casters, two laser range finders, and a tilt sensor and controller.

Other methods to assist and/or monitor the patient's physical health include: robo-cane [46], accelerometer-based gait analysis [47], camera-based posture and gait analysis [48]. These and other studies will be further explained in Section 6.

### 5.3. Psychological Factors

Psychological risk factors are those factors that alter a person's cognition, either temporarily or permanently. Cognition is a mental process that deals with how a person understands and behaves in the world. When a person's cognitive faculties are not fully functional, the simplest tasks become extremely complex and hard to complete [49].

When a person experiences a fall due to psychological factors, it may be because one or more brain cognitive functions fail to perform their job. These are the cognitive brain functions that have a direct impact on fall-related events: perception, attention, motor, visual and spacial processing, and executive functions. Perception is the one in charge of recognition and interpretation of sensor stimuli (e.g., touch, hearing, smell, *etc.*). Attention deals with the ability to maintain concentration on a specific object, action, and/or thought. The motor brain function controls the ability to move limbs and extremities. Visual and spacial processing manage the ability to process incoming visual stimulus, and interpret how objects interact with one another in terms of the spacial relations among them. The executive brain function permits people to make plans and execute them [49].

Emotions are strong factors that may significantly impact health in a number of ways. They can be highly overwhelming affecting mental state, ability to be optimistic about life, and/or self perception. Depression, loneliness, and anxiety are some of the many psychological emotion-related factors that contribute to fall accidents. Peel *et al.* [50] conducted a study involving 761 participants age 65 or older, where the main objective was to evaluate the relationship between psychological factors and falls among the elderly in Baghdad city, Iraq. Their main finding is that depression is the most important risk factor that contributes to fall accidents.

An important area of research, yet ignored, is the influence of psycho-social factors in fall-related injuries [50]. They are considered to have a major role in aging. The following are some of these factors: stress and coping, marital status, living arrangements, life satisfaction, emotional status, cognition and social correctness [50]. Factors with a negative connotation may produce negative symptoms such as lack of air and/or dizziness. Peel *et al.* [50] conducted a study with 387 participants to determine the contribution of psycho-social factors to fall-related hip fractures. The data collection questionnaires were administered through face-to-face interviews. Three psycho-social factor domains were defined, namely community support systems, psychological well-being, and engagement with life. Some of the results of this study are the following:

- Marital status is a determinant of fall-related hip fractures due to the beneficial effects of marriage on health behaviors. Life partners look after each other.
- Living alone represents a greater vulnerability to an unhealthy lifestyle and a reduced social network, which are tightly related to fall related hip fractures.
- Depression is one of the biggest fall-related accident triggers.

## 6. Evaluation of The Systems

In order to evaluate the state-of-the-art FP and FD systems, it is necessary to establish a common ground where the systems can be compared and analyzed. This paper presents a qualitative evaluation of the most important FD and FP systems based on the design issues described in Section 3.

The criteria used to correlate and assess each system is based on the following elements:

- Type of sensor used (external or wearable)
- Feature analyzed (Gait, posture, *etc.*)
- Falling Factors (Section V)
- Level of obtrusiveness (Low, Medium, High)
- Energy Consumption (Low, Medium, High)
- Data Collection scenario
- Learning Algorithm
- Overall accuracy

Albeit a large number of systems are present in the literature, twenty four have been selected based on their relevance in terms of the aforementioned elements.

The abbreviations and acronyms used are defined in Table 3. Table 4 summarizes the state-of-the-art fall detection approaches. Table 5 reviews the state-of-the-art fall prevention systems.

### 6.1. Fall Detection Systems

The most relevant studies on fall detection systems are described next.

#### 6.1.1. Bilgin *et al.*

This system [51] uses a data mining approach to detect falls. It is based on wireless sensor networks (WSNs). A WSN is a network where all of its nodes (also known as motes) are deployed in predefined locations gathering data about their surrounding. Each mote sends the collected information wirelessly (RF) to a base station or sink where the information is processed. Motes are usually equipped with multiple types of sensors (e.g., thermometers, barometers, microphones, accelerometers, *etc.*). Motes are constrained in energy, computational power and bandwidth. This work only employs the accelerometer, and it is only capable of detecting a fall when a person is standing. The sensor is placed on the waist of the subject. It uses a k-NN (Section II) approach to detect if a fall has occurred. The accuracy obtained on the conducted experiments was 89.4% with a recall of 100% and precision of 85%.

#### 6.1.2. Ozcan *et al.*

The basic idea behind this solution [10] is to create an autonomous system that is able to provide quick and accurate real-time responses to critical events like a fall, while preserving computational resources. The system is not only able to detect falls but also to classify non-critical events such as sitting and lying down. Their solution is based on a modified version of the histogram of oriented gradients (HOGs) algorithm [67]. When a fall occurs edge orientations in a frame vary drastically and extremely fast, as a result of this subsequent frames get blurred. A dissimilarity distance is computed between two frames, and if it is greater than a predefined threshold a fall is detected. A microsoft LifeCam camera mounted on the center of the SOI's pelvis was used to capture the images. One of the drawbacks of the system is its lack of auto exposure adjustment in the camera. False alarms may be raised if the scenery changes.

#### 6.1.3. Bashir *et al.*

The proposed system [53] is based on a wireless body area network. It uses a tri-axial accelerometer, and a tri-axial gyroscope embedded in a necklace-like sensor. Three stages are used to determine human status namely, fall, activity, and sleep. The information is sent to a base station that receives and stores the data. The algorithms used are threshold-based and very simple. It employs the posture angle, angular velocity, and acceleration to determine if a fall has occurred. If the aforementioned features surpass a pre-established threshold a fall is detected. The accuracy for ADLs was 100%, while the overall recall was 81.6%.

#### 6.1.4. PerFallD

This system [54] was one of the first, if not the first, smartphone android-based fall detection system. It was introduced by Dai *et al.*, in 2010. PerFallD takes advantage of the sensors (e.g., accelerometer, gyroscope, *etc.*) and communication technologies embedded in a smartphone to not only detect falls, but also to send alerts automatically when a patient is not responsive within a pre-specified time after a fall. Its user interface is highly friendly, and no additional hardware is required. The acceleration and angular data are used to set thresholds that determine if a fall has occurred. People nowadays are used to carrying their cellphones everywhere, making PerfallD highly unobtrusive. Multiple experiments were performed placing the phone in different locations (chest, waist and thigh); different thresholds had to be set per location. The waist was found to be the best location with an average performance of 2.76% for FN and 8.7% for FP.

#### 6.1.5. uCare

uCare [55] is another fall detection system based on an android-based smartphone. It was developed by Shi *et al.*, in 2012, and it integrates a support vector machine (SVM) (Section 2) in the classification stage. The proposed technique uses the acceleration data from the phone's accelerometer to detect a fall. The fall detection process is divided into five phases namely, normal, unstable, free fall, adjustment, and motionless. Normal refers to ADLs. Unstable is related with the moment prior the fall. Free fall is experimented when falling. Adjustment deals with the impact of the fall. Motionless is the period of time after the fall when the subject is not moving. An acceleration threshold is used to trigger the five-phases feature extraction method. A 16-elements vector is obtained as a result of the extraction method. This vector is fed into a SVM that is used to differentiate falls from ADLs. The data collection and experiments were conducted in a lab environment using a Google Nexus S phone and MATLAB was used to build the classifier. The acquired results were the following: recall 90% and precision 95.7%.

#### 6.1.6. eHome

Werner *et al.*, developed an ambient assisted living system (AAL) that thrives for giving old people the feeling of security in their homes. The prototype was created in 2011 and was named eHome [56]. The idea behind ehome is to provide a user's home with a set of unintrusive wireless sensors to gather his/her behavioral data when faced to a life-threatening situation such as a fall. The system uses floor-mounted accelerometers to collect vibration patterns of an individual that are sent wirelessly to a base station where data analysis and decision-making is accomplished. The fall detection process is based on the belief that distinctive impact vibrations propagate through the floor when a fall occurs. These vibrations are compared to a threshold in order to identify if a fall-related accident took place. When a fall is detected the user is asked if he/she is ok or not. If the user does not respond within a predefined period an alarm is triggered and relatives and care givers are notified. eHome uses at least three sensor boxes that are placed in the middle of the room's edges; the boxes are within an aggregated network. The Fast Fourier Transform is one of the techniques used for feature extraction. The initial experiments were performed in a lab environment and later on in real home settings. No real fall happened in the real trials, as a result no conclusion for this scenario was provided. The recall and specificity in the experiments were 87% and 97%, respectively.

#### 6.1.7. Sengto *et al.*

In 2012, Sengto *et al.* proposed a fall detection system algorithm based on a back propagation neural network (BPNN) [57]. The system utilizes a tri-axial accelerometer mounted on the user's waist in order to collect his/her acceleration data behavior. Human activities are divided in three groups: falling activities (forward, backward, right and left), slow motion activities (walking, getting up from bed, flopping), and sudden motion activities (running, jumping). An acceleration threshold is set to differentiate between slow motion activities and other activities. Sudden motion and falling activities have similar acceleration signal patterns. If the threshold is surpassed a BPNN will determine if a fall occurred. The overall recall of the detection algorithm was 96.25% while the specificity was 99.5%.

#### 6.1.8. Chen *et al.*

A human fall detection system using a computer vision approach is introduced in [29]. The solution is capable of detecting fall-related events in real time using skeleton features and human shape variations. The system is able to extract the human posture and reduce the computational burden by using a 2D model instead of a complicated 3D one. The human contour is partitioned into triangular meshes by using the constrained delaunay triangulation algorithm [68]. The skeleton (a spanning tree) is acquired by running the well-known graph traversal algorithm Depth-first search (DFS) on the center of the triangular meshes. The Douglas-Peucher algorithm is used to reduce the number of pixel in the human contour, effectively decreasing the computational complexity. A distance map is used to calculate the distance between two skeletons. A fall is detected if the user's motion does not change within a certain period of time. The system is able to obtain a high detection accuracy (90.9%) while maintaining a low false alarm rate.

#### 6.1.9. Yu *et al.*

A posture recognition-based fall detection system is proposed in [9]. It uses computer vision techniques to detect fall-related accidents. Background subtraction is applied to distinguish moving objects from the background. The resulting image is polished in postprocessing to get a more accurate body silhouette. Feature extraction is achieved through ellipse fitting and a projection histogram. A multiclass support vector machine (DAGSVM) is used for classification purposes. Ground detection is also performed to improve the accuracy of the system. Three conditions are necessary to detect a fall: the posture is classified as “lie” or “bend”, the posture is inside the ground region, and the first two conditions must be held for 30 s. The system suffers from occlusion and multiple moving objects. The experiments showed promising results with a detection rate of 97.08% and a low false detection rate of 0.8%.

#### 6.1.10. Sorvala *et al.*

False alarms can represent a major issue for nurses and physicians who have to take care of several patients in the ICUs [41]. Sorvala *et al.*, developed a fall detection algorithm that reduces false alarms. The algorithms employs a tri-axial accelerometer, a tri-axial gyroscope (both mounted on the pelvis of the patient), and an ankle-worn tri-axial accelerometer. The solution distinguishes from falls, possible falls, and ADLs. A possible fall is one where the subject gets up within a 10-second period after the detected impact. If the subject does not recover within the specified time an alarm will be sent to a predefined person (nurse, doctor, relative). It uses a ruled-based approach to differentiate from the aforementioned activities. The total sum vector magnitude for the tri-axial acceleration data and the total sum vector magnitude for the tri-axial angular velocity are used to determine if there was an impact. The use of a two-threshold algorithm showed that this system is better suited to detect falls than the ones that only use one.

#### 6.1.11. Humenberger *et al.*

In 2012 a bio-inspired stereo vision fall detection system was introduced by Humenberger *et al.* [58]. The system design is described in [69] and it utilizes two optical detector chips, a field-programmable gate array (FPGA), a digital signal processor (DSP) and a wireless communication module. The optical chips capture video frames. The FPGA creates the input data for the DSP by calculating 3D representations of the environment. The DSP is loaded with a neural network that is used for classification purposes. Falls are divided into 4 states or phases pre-fall, critical, post-fall, and recovery phase. To run the experiments the hardware was mounted on the top corners of a room in order to monitor the subjects of interest. The trial results are 90% of fall detection rate for all networks, and 97%–98% for the best network.

#### 6.1.12. Chen *et al.*

Chen *et al.* proposed a wireless fall detector using accelerometers [59]. The system consists of two modules, a fall detection terminal and a remote one; they can communicate with each other through wireless communication. The fall detection terminal works without the remote one. The remote terminal is used to receive and store kinematic characteristics and raise alarms. The solution employs an inertial sensor (an accelerometer), a microcontroller and wireless SoC (System on chip). A threshold-based algorithm is used to detect falls. Five tests were performed: forward fall (FF), backward fall (BF), left-side fall (LF), right-side fall (RF), and ADLs. The results of the study showed a 97% of recall and 100% of specificity.

#### 6.1.13. Yuwono *et al.*

This system uses a gaussian distribution of clustered knowledge, an augmented radial basis neural network (ARBFNN) and a multilayer perceptron (MLP) to detect falls [60]. 3D acceleration data collected from the user's waist is used as the input signal. The collected information is sent to a receiver board via wirelessly. If the acceleration magnitude surpasses a predefined threshold the input signal is pushed to a classification queue. A discrete wavelet transform (DWT) is employed to filter the acceleration signal and reduce the sampling signal rate; decreasing complexity and improving classifier generalization. The filtered data are fed into an ensemble classifier: MLP neural network and an ARBFNN. Falls and ADLs were simulated by volunteers in a lab environment using mattresses. The results of the experiment are the following: an ingroup recall of up 100%, an outgroup recall of 97.65% and an ADLs specificity of 99.33%.

#### 6.1.14. SAFE

In 2011 a fall detection system using wearable sensors was introduced by Ojetola *et al.* [61]. The system uses a wireless sensor platform known as SHIMMER (Sensing Health with Intelligence, Modularity, Mobility and Experimental Reusability) [70]. Every SHIMMER node is equipped with a tri-axial accelerometer and tri-axial gyroscope, a bluetooth device and a microcontroller device. Two SHIMMER nodes were used, one in the CHEST and the other one in the subject's right thigh. The acceleration and angular velocity magnitudes were used as input features for a C4.5 decision tree (Section 2). Four fall types were aimed to be detected, namely forward, backward, right and left. Falls and ADLs were simulated by volunteers in a lab environment. Leave one-out and leave N-out methods were used to evaluate the system performance. Falls and ADLs were identified with a precision of up to 81% and recall of 92%. The accuracy ranged from 98.5% to 99.45%.

#### 6.1.15. Shoaib *et al.*

A context-based fall detection method was presented in 2010 by Shoaib *et al.* [62]. It exploits computer vision techniques in order to identify head and feet locations to build a context model for detecting falls. A fall is detected by comparing the position of the head from the floor. A combination of ellipse matching and skin color matching is used to detect the head of the subject in the frames of a video clip. Once the head has been located, the feet area is found using the medial axis [71]. Frames are divided into three blocks, namely head, floor and neutral blocks. Three conditions are necessary to detect a fall, head location, feet location, and the vertical distance of the head from the head centroid mean. Six fall types are considered, backward, forward, lateral (right and left), syncope (fainting) and neutral. The system was tested using twenty six video clips recorded in real home environments. The reported accuracy was 96%.

### 6.2. Fall Prevention Systems

The most relevant studies on fall prevention systems are described next.

#### 6.2.1. Rantz *et al.*

Rantz *et al.* proposed an in-home monitoring system that monitors the daily routine of a person and collects gait-related data for fall risk assessment [48]. The system is able to detect when a fall occurs or when the risk of falling increases. A pulse-Doppler range control radar, a Microsoft kinect, and two web cams were used to track the patient's movements inside his/her house. This information is analyzed and periodic evaluations of the patient's gait conditions are made. If a change from the normal gait parameters are detected an alarm is raised, the patient and care givers are notified. To preserve the patient's privacy only the depth image was used. The data collection process took eighteen months, and the system was deployed in an independent senior living community. The ground truth was provided by a vicon optical montion capture system that uses infrared markers that were worn by the SOI's; monthly fall risk assessments were performed by the health care provider to validate and improve the sensor system. The results were promising since high correlation between the ground truth and the deployed system was found.

#### 6.2.2. Fallarm

Vision based systems perform properly only if the whole environment is equipped with cameras [8]. Besides being expensive, they may fail when more than one subject is in the field of view. Fallarm is a pervasive fall prevention system meant to be used in hospitals and care facilities. The hardware consists of an un-obtrusive wearable wrist device, a visual feedback component, an auditory feedback component (mini speakers), and a tactile feedback component. Mounted on the wrist device, an inertial sensor is used to extract the mobility patterns of the subject. The system keeps the user informed about his/her current falling risk level through a traffic-light-like alert system via the visual feedback component. Three different colors are used to represent the risk of falling: red, amber, and green to indicate high, medium and low respectively. When the risk level rises the tactile feedback component is activated. The user receives a short ascending pattern of vibrations by means of a pager motor. Conversely, if the risk level decreases a short descending pattern of vibrations will be generated. If the user persists in performing activities the system identifies as dangerous a continuous vibration will be triggered in the pager. In addition to these functions, the care provider will be notified to assess the situation and take the corrective measures.

#### 6.2.3. Hirata *et al.*

Hirata *et al.* [30] proposed a method that calculates the user's center of gravity position based on the user's skeleton model (human model) in the sagittal plane [13]. The system uses passive-type walkers for fall prevention. Two conditions were considered for the creation of the human model. First the user must hold the walker with both hands. Second, the user's limbs (legs and arms) must be measured a priori. Two laser range finders were used, one placed at the same height of user's hip joint, and the other one at the lower part of the walker. The first one is used to create the upper section of the skeleton, and the second one for the lower part. The center of gravity of the human model is used to determine whether the user is within a predefined stability region. If the user is outside the stability region the servo brakes will be triggered making the walker stop. As the walker stops, the user has a better support to accommodate himself/herself and return to the stability region.

#### 6.2.4. Ni *et al.*

The majority of the patients that suffer a fall while in the hospital, tried to either suddenly get up from their bed or move from the bed to a chair [63]. Ni *et al.* designed a computer-vision-based system to prevent in-hospital patients from falling from their beds. This system performs event detection, specifically, detecting when a person gets up from a bed. The idea behind this study is to alert the nursing staff when the patient gets up. In this way, nurses can immediately assist him/her and avoid any fall. A Microsoft kinect was used to capture the images of the patients. The camera is mounted to a bed from the side view. A specific region of interest was used (the bed area), that was further divided into 4 × 2 equally sized rectangular boxes, which were called channels. A multiple kernel learning framework was employed to extract the features from the channels. A leave-one-subject-out testing scheme was utilized to measure the performance of the system. The system was compared with a state-of-the-art recognition system (STIPs [72]), which was outperformed by its results.

#### 6.2.5. Takeda *et al.*

Takeda *et al.* developed a foot age assessment system that estimates how likely a person is to fall based on his/her balance ability and gait condition [45]. The proposed method employs a person's age to infer his/her probability of suffering a fall. The system uses mat type distribution sensor to gather the SOI's gait characteristics. Through fuzzy logic the system is able to make educated guesses about the SOI's age which is supposed to have a correlation with falling risks. The fuzzy membership functions were obtained through a learning process. The system was evaluated through leave the one-out cross validation method. Although not very reliable, this system is able to shed some light on how age and gait condition are related.

#### 6.2.6. iCane

Rehabilitation robots are another way to help the elderly to overcome their mobility problems [46]. These robots are commonly used for assisting and training the whole body. Di *et al.* designed an omnidirectional cane-type robot called iCane that helps old adults to walk. The system is considered a smart-cane since it is able to not only assist people to walk but also to prevent and detect falls. The idea behind this cane is to perform optimized actions for the user such as guiding, fall prevention, rehabilitation, and much more. The concept of intentional direction (ITD) is introduced, where the robot estimates the direction where the person wants to move to. The intelligent cane was constructed with the following elements: a force sensor, an omni-directional mobile base, a metal stick, and a laser rangefinder (LRF). The system uses the center of gravity (COG) of the user in conjunction with the cane sensors to create a fall prevention strategy.

#### 6.2.7. Hsieh *et al.*

Hsieh *et al.* proposed a falling characteristics collection system that gathers patients' fall patterns in order to create fall prevention strategies [43]. The prototype takes advantage of a 6G tri-axial accelerometer that measures the three axial accelerations of a fall. The device monitors the whole body movements, and it is placed on the SOI's pelvis due to its proximity to the center of the body mass. The detection algorithm that was proposed in [40] was used to detect falls. The results of this study can be used to find relationships between falls and particular injuries.

#### 6.2.8. smartPrediction

In 2013, Majumber *et al.* presented smarPrediction, a real time fall prediction and prevention platform [64]. The system is composed of a smartphone and a smartshoe. The accelerometer and the gyroscope embedded in the phone, along with four pressure sensors placed at the bottom of a shoe are used to collect gait/walking data of the user. The smartphone and the smartshoe are connected via wifi. The system uses an android-based application that is capable of triggering an alarm when an anomaly in the subject's gait condition is detected. A decision tree with 10 fold-cross validation was used to validate the effectiveness of the implemented approach. A 97.2% accuracy was found in gait abnormality detection.

#### 6.2.9. STRATIFY

St Thomass' risk assessment tool in falling elderly inpatients (STRATIFY) is a risk assessment tool developed to predict elderly patient falls in hospitals [65]. It uses a 0–5 (low risk–high risk) score system to rank patients' likelihood of experiencing a fall. Patients of elderly units from St Thomas, Kent and Canterbury Hospital in the UK were monitored in order to identify the risk factors of a fall. After a fall occurs a series of falling characteristics (risk factors) are recorded in a log and used to compute the probability of a future fall. Five factors were found to have a direct impact in falls and were used to build the risk assessment tool. These factors were agitation, patient was admitted with a fall or patient fell inside the ward, visual impairment, need of toileting, and a transfer and mobility score (0–6) based on the transfer and mobility section of the barthel index [73]. The recall and specificity of the score for fall prediction were 92% and 68% respectively.

## 7. Conclusions

This paper surveys the state-of-the-art fall detection and prevention systems. In order to give a basic understanding of the underlying concepts employed in such systems their general model is presented and its modules are explained; the data collection module is in charge of gathering the user's motion data; the feature extraction module selects relevant and more meaningful characteristics that are fed into the learning module; the Learning module is used to find relationships from the extracted features in the training set. As a result of this process, a descriptive model is generated; the evaluation model assesses the performance of the generated model. The most important aspects that need to be considered when designing FD and FP systems are described; these design issues are: obtrusiveness, occlusion, multiple people in the scene, aging, privacy, computational cost, energy consumption, noise, and defining a threshold. A three-level taxonomy (physical, psychological and environmental) is used to describe the falling risk factors associated with a fall. Finally, cutting edge FD and FP approaches are thoroughly reviewed and qualitatively compared. This evaluation contrasts the associated challenges and design issues these systems face. It serves as a template where all systems are forced to fit, making it easier to establish correlations among them. It provides the tools to objectively decide which system is better suited for any given scenario.

## Figures and Tables

**Table 1.** Example of a Dataset.

**Outlook**	**Temperature**	**Humidity**	**Windy**	**Play**
Sunny	Hot	High	False	No
Overcast	Hot	High	True	Yes
Rainy	Mild	Normal	False	No
Rainy	Cool	Low	False	No
Overcast	Cool	Normal	True	Yes
Overcast	Mild	High	True	Yes
Rainy	Mild	High	True	No

**Table 2.** Comparison among supervised learning algorithms.

**Algorithm**	**Data Structure**	**Approach**	**Time Complexity**
Decision Tree	Trees	Divide And Conquer	*O*(*m* · *n* · *log n*)+ *O*(*n* · (*log n*)^2^)
Naive Bayes	Matrices	Probabilistic Straightforward	*O*(*T* · *A*)
K-nearest Neighbor	Matrices	Brute Force	*O*(*n*^2^)
Support Vector Machine	Matrices	Optimization	(Primal) *O*(*n* · *a*^2^ + *a*^3^) Dual (*O*(*a* · *n*^2^ + *n*^3^)

**Table 3.** List of Abbreviations and Acronyms.

**Abbreviation**	**Description**	**Abbreviation**	**Description**
3D-ACC	Tri-axial Accelerometer	IP	Inactivity Period
3D-GYR	Tri-axial Gyroscope	KNT	Microsoft Kinect
AC	Accuracy	k-NN	k-Nearest Neighbor
ADL	Activity of Daily Living	LDM	Load Distribution Sensor Mat
ARBFNN	Augmented Radial Basis Neural Networks	LRFs	Laser Rangefinders
BPNN	Back Propagation Neural Networks	MLP	Multilayer Perceptron
BSLT	Body Silhouette	OCSVM	Online one-class Support Vector Machine
COG	Center of Gravity	PDRCR	Pulse-Dopler Range Control Radar
CM	Color Matching	PHY	Physical
DAGSVM	Directed Acyclic Support Vector Machine	PR	Precision
DT	Decision Tree	PRSS	Pressure Sensor
EM	Ellipse Matching	PSY	Psychological
ENV	Environmental	PST	Posture
FS	Falling Signal	RC	Recall
FSE	Force Sensor	SCH	Sudden Change
FTDNN	Focused Time Delay Neural Network	SP	Specificity
HI	Head Information	SVMs	Support Vector Machines
HP	Highest Point of the Body	TB	Threshold Based
HS	Human Skeleton	VC	Video Camera
IN	Inertial Sensor	VM	Vibration Magnitude

**Table 4.** Summary of state-of-the-art fall detection systems.

**Reference**	**Experiment Environment**	**Sensor**	**Sensor's Location**	**Feature Analyzed**	**Obtrusive**	**Energy Consumption**	**Cost**	**Comp. Complexity**	**Learning**	**RC / PR / SP / AC**
Bilgin [51]	LAB	3D-ACC	WAIST	SCH + IP	MEDIUM	MEDIUM	MEDIUM	LOW	k-NN	100% / 85% / NA / 89.40%
Ozcan [52]	LAB	VC	SUBJECT'S VICINITY	PST	MEDIUM	HIGH	HIGH	HIGH	TB	NA / NA / NA / 84% 86%
Bashir [53]	LAB	3D-ACC + 3D-GYR	NECK	PST	MEDIUM	LOW	LOW	LOW	TB	81.60% / NA / NA / NA
PerFallD [54]	LAB	SMARTPHONE (3D-ACC + 3D-GYR)	WAIST (BELT)	PST	LOW	LOW	MEDIUM	LOW	TB	NA / 91.3% / NA / NA
uCare [55]	LAB	SMARTPHONE (3D-ACC)	POCKET	SCH + IP	LOW	LOW	MEDIUM	LOW	TB + SVMs	90% / 95.7% / NA / NA
eHome [56]	LAB + REAL HOME	3D-ACC	FLOOR	VM + IP	LOW	HIGH	MEDIUM	LOW	TB	87% / NA / 97.7% / NA
Sengto [57]	LAB	3D-ACC	WAIST	FS	MEDIUM	LOW	LOW	LOW	BPNN	96.25% / NA / 99.50% / NA
Chen [29]	LAB	VC	SUBJECT'S VICINITY	HS	LOW	HIGH	HIGH	HIGH	TB	NA / NA / NA / 90.09%
Yu [9]	LAB	VC	SUBJECT'S VICINITY	BSLT	LOW	HIGH	HIGH	HIGH	DAGSVM	NA / 99.2% / NA / 97.08%
Sorvala [41]	LAB	SMARTPHONE (3D-ACC + 3D-GYR)	WAIST AND ANKLE	SCH + IP	LOW	LOW	MEDIUM	MEDIUM	TB	95.60% / NA / 99.6% / NA
Humenberger [58]	LAB	VC	SUBJECT'S VICINITY	COG + HP + BSLT	LOW	HIGH	HIGH	HIGH	FTDNN	NA / NA / NA / 90% - 99%
Chen [59]	LAB	3D-ACC	WAIST	SCH + IP	MEDIUM	LOW	LOW	LOW	TB	97% / NA / 100% / NA
Yuwono [60]	LAB	3D-ACC	WAIST	FS	MEDIUM	MEDIUM	LOW	HIGH	TB + ENSEMBLE CLASSIFIER (MLP + ARBF)	97.65% / NA / 96.59% / NA
SAFE [61]	LAB	SMARTPHONE (3D-ACC + 3D-GYR)	CHEST AND THIGH	FS	HIGH	MEDIUM	MEDIUM	LOW	DT	92% / 81% / NA / 98.91% - 99.45%
Shoaib [62]	REAL HOME	VC	SUBJECT'S VICINITY	HI + FI	LOW	HIGH	MEDIUM	HIGH	TB +EM+CM	NA / NA / NA / 96%

**Table 5.** Summary of state-of-the-art fall prevention systems.

**Reference**	**Experiment Environment**	**Sensor**	**Sensor's Location**	**Taxonomy**	**Feature Analyzed**	**Obtrusive/ Invasive**	**Energy Consumption**	**Cost**	**Computational Complexity**
Rantz [48]	LAB + COMMUNITY	PDRCR + KNT	SUBJECT'S VICINITY	PHY + PSY	GAIT CONDITION	LOW	HIGH	HIGH	HIGH
Fallarm [8]	LAB + CLINICAL FACILITY	IN	WRIST	PHY	MOBILITY PATTERNS	LOW	LOW	LOW	LOW
Hirata [30]	LAB	LRFs	WALKER	PHY + PSY	DISTANCE BTW USER AND WALKER	HIGH	MEDIUM	MEDIUM	LOW
Ni [63]	HOSPITAL	KNT	SUBJECT'S VICINITY	PSY	POSTURE	LOW	HIGH	MEDIUM	HIGH
Takeda [45]	LAB	LDM	FLOOR	PHY + PSY	SOLE PRESSURE	LOW	MEDIUM	MEDIUM	MEDIUM
iCane [46]	LAB	FSE + LRFs	CANE	PHY	GAIT CONDITION	MEDIUM	HIGH	HIGH	MEDIUM
Hsieh [43]	LAB	3D-ACC	WAIST	PHY	FALLING DIRECTION AND IMPACT PARTS	HIGH	LOW	LOW	LOW
smartPrediction [64]	LAB	SMARTPHONE (3D-ACC + 3D-GYR) + PRSS	POCKET + SHOE	PHY	GAIT CONDITION	HIGH	MEDIUM	MEDIUM	MEDIUM
STRATIFY [65,66]	HOSPITAL	NA	NA	PHY +PSY+ ENV	AGE BARTHEL INDEX USE OF WALKING AID CURRENT MEDICATION VISION NURSES' JUGDEMENT	LOW	NA	LOW	NA

## References

[1] Nihseniorhealth: About falls. http://nihseniorhealth.gov/falls/aboutfalls/01.html.

[2] Nihseniorhealth: Causes and Risk Factors. http://nihseniorhealth.gov/falls/causesandriskfactors/01.html.

[3] Lord S.R., Menz H.B., Sherrington C. (2006). Home environment risk factors for falls in older people and the efficacy of home modifications. Age Ageing.

[4] Health: 10 Ways to Prevent Falls at Home. http://www.health.com/health/gallery/0,20364937_2,00.html.

[5] News & Information: Healthy Aging Column—Preventing Falls. http://www.news.colostate.edu/Release/452.

[6] CDC: Preventing Falls among Older Adults. http://www.news.colostate.edu/Release/452.

[7] Nihseniorhealth: Fall Proofing Your Home. http://nihseniorhealth.gov/falls/homesafety/01.html.

[8] Caporusso N., Lasorsa I., Rinaldi O., la Pietra L. A pervasive solution for risk awareness in the context of fall prevention.

[9] Yu M., Rhuma A., Naqvi S.M., Wang L., Chambers J. (2012). A posture recognition based fall detection system for monitoring an elderly person in a smart home environment. IEEE Trans. Inf. Technol. Biomed..

[10] Ozcan K., Mahabalagiri A., Casares M., Velipasalar S. (2013). Automatic fall detection and activity classification by a wearable embedded smart camera. IEEE J. Emerg. Sel. Top. Circuits Syst..

[11] Kim E., Helal S., Cook D. (2010). Human activity recognition and pattern discovery. IEEE Pervasive Comput..

[12] Lara O.D., Labrador M.A. (2013). A survey on human activity recognition using wearable sensors. IEEE Commun. Surv. Tutor..

[13] Hirata Y., Komatsuda S., Kosuge K. Fall prevention control of passive intelligent walker based on human model.

[14] Yu X. Approaches and principles of fall detection for elderly and patient.

[15] López J.M., Herrero J.G. (2006). Técnicas de análisis de datos. Aplicaciones Prácticas Utilizando Microsoft Excel y WEKA.

[16] Sapsford R., Jupp V. (2006). Data Collection and Analysis.

[17] Guyon I., Elisseeff A., Guyon I., Nikravesh M., Gunn S., Zadeh L. (2006). An Introduction to Feature Extraction. Feature Extraction.

[18] Witten I.H., Frank E., Hall M.A. (2011). Data Mining: Practical Machine Learning Tools and Techniques.

[19] Mayfield E., Penstein-Rosé C. Using feature construction to avoid large feature spaces in text classification.

[20] Turaga P., Chellappa R., Subrahmanian V., Udrea O. (2008). Machine recognition of human activities: A survey. IEEE Trans. Circuits Syst. Video Technol..

[21] Quinlan J.R. (1993). C4.5: Programs for Machine Learning.

[22] Campbell C., Cristianini N. (1998). Simple Learning Algorithms for Training Support Vector Machines.

[23] Chapelle O. (2007). Training a support vector machine in the primal. Neural Comput..

[24] Gunawardana A., Shani G. A (2009). Survey of accuracy evaluation metrics of recommendation tasks. J. Mach. Learn. Res..

[25] Szeliski R. (2010). Computer Vision: Algorithms and Applications.

[26] Gasparrini S., Cippitelli E., Spinsante S., Gambi E. A depth-based fall detection system using a Kinect sensor. Sensors.

[27] Lara O.D. (2012). On the Automatic Recognition of Human Activities Using Heterogeneous Wearable Sensors. Ph.D. Thesis.

[28] Lent B. (2007). Practical Considerations of Accelerometers Noise.

[29] Chen Y.T., Lin Y.C., Fang W.H. A hybrid human fall detection scheme.

[30] Hirata Y., Muraki A., Kosuge K. Motion control of intelligent passive-type walker for fall-prevention function based on estimation of user state.

[31] Williams A., Ganesan D., Hanson A. Aging in place: Fall detection and localization in a distributed smart camera network.

[32] Vaidehi V., Ganapathy K., Mohan K., Aldrin A., Nirmal K. Video based automatic fall detection in indoor environment.

[33] Rougier C., Meunier J., St-Arnaud A., Rousseau J. Fall detection from human shape and motion history using video surveillance.

[34] Wong W.K., Lim H.L., Loo C.K., Lim W.S. Home alone faint detection surveillance system using thermal camera.

[35] Alwan M., Rajendran P.J., Kell S., Mack D., Dalal S., Wolfe M., Felder R. A smart and passive floor-vibration based fall detector for elderly.

[36] Sixsmith A., Johnson N., Whatmore R. (2005). Pyroelectric IR sensor arrays for fall detection in the older population. J. Phys..

[37] Sixsmith A., Johnson N. (2004). A smart sensor to detect the falls of the elderly. IEEE Pervasive Comput..

[38] Scott T. Bed Exit Detection Apparatus. http://www.google.com/patents/US6067019.

[39] Haselman M., Hauck S. (2010). The future of integrated circuits: A survey of nanoelectronics. Proc. IEEE.

[40] Chen G., Huang C., Chiang C., Lee Y., Bien Z.Z., Mokhtari M., Kim J.T., Park M., Kim J., Lee H., Khalil I. (2010). A reliable Fall Detection System Based on Wearable Sensor and Signal Magnitude Area for Elderly Residents. Aging Friendly Technology for Health and Independence.

[41] Sorvala A., Alasaarela E., Sorvoja H., Myllyla R. A two-threshold fall detection algorithm for reducing false alarms.

[42] Vallejo M., Isaza C.V., Lopez J.D. Artificial neural networks as an alternative to traditional fall detection methods.

[43] Hsieh C.J., Chiang C.Y., Huang C.N., Chan C.T., Hsu S.J. Development of fall characteristics collection system for fall prevention strategies.

[44] Zweifel P., Felder S., Meiers M. (1999). Ageing of population and health care expenditure: A red herring?. Health Econ..

[45] Takeda T., Sakai Y., Kuramoto K., Kobashi S., Ishikawa T., Hata Y. Foot age estimation for fall-prevention using sole pressure by fuzzy logic.

[46] Di P., Huang J., Sekiyama K., Fukuda T. A novel fall prevention scheme for intelligent cane robot by using a motor driven universal joint.

[47] Shiozawa N., Arai S., Okada S., Makikawa M. Gait analysis of sit-to-walk motion by using portable acceleration monitor device for fall prevention.

[48] Rantz M.J., Skubic M., Abbott C., Galambos C., Pak Y., Ho D.K.C., Stone E.E., Rui L., Back J., Miller S.J. (2013). In-home fall risk assessment and detection sensor system. J. Gerontol. Nurs..

[49] Sharpbrains: What Are Cognitive Abilities and Skills, and How to Boost Them?. http://sharpbrains.com/blog/2006/12/18/what-are-cognitive-abilities/.

[50] Peel N.M., McClure R.J., Hendrikz J.K. (2007). Psychosocial factors associated with fall-related hip fractures. Age Ageing.

[51] Bilgin T., Erdogan S. (2012). A data mining approach for fall detection by using k-nearest neighbour algorithm on wireless sensor network data. IET Commun..

[52] Yu M., Yu Y., Rhuma A., Naqvi S.M.R., Wang L., Chambers J.A. (2013). An online one class support vector machine-based person-specific fall detection system for monitoring an elderly individual in a room environment. IEEE J. Biomed. Health Inf..

[53] Bashir F. Real life applicable fall detection system based on wireless body area network.

[54] Dai J., Bai X., Yang Z., Shen Z., Xuan D. PerFallD: A pervasive fall detection system using mobile phones.

[55] Shi Y., Shi Y.C., Wang X. Fall detection on mobile phones using features from a five-phase model.

[56] Werner F., Diermaier J., Panek P., Schmid S. Fall detection with distributed floor-mounted accelerometers: An overview of the development and evaluation of a fall detection system within the project eHome.

[57] Sengto A., Leauhatong T. (2012). Human falling detection algorithm using back propagation neural network.

[58] Humenberger M., Schraml S., Sulzbachner C., Belbachir A.N., Srp A., Vajda F. Embedded fall detection with a neural network and bio-inspired stereo vision.

[59] Chen D., Feng W., Zhang Y., Li X., Wang T. A wearable wireless fall detection system with accelerators.

[60] Yuwono M., Su S.W., Moulton B. Fall detection using a gaussian distribution of clustered knowledge, augmented radial basis neural-network, and multilayer perceptron.

[61] Ojetola O., Gaura E.I., Brusey J. Fall detection with wearable sensors–SAFE (Smart Fall DEtection).

[62] Shoaib M., Dragon R., Ostermann J. View-invariant fall detection for elderly in real home environment.

[63] Ni B., Nguyen C., Moulin P. RGBD-camera based get-up event detection for hospital fall prevention.

[64] Majumder A.J.A., Zerin I., Uddin M., Ahamed S.I., Smith R.O. smartPrediction: A real-time smartphone-based fall risk prediction and prevention system.

[65] Oliver D., Britton M., Seed P., Martin F.C., Hopper A.H. (1997). Development and evaluation of evidence based risk assessment tool (STRATIFY) to predict which elderly inpatients will fall: Case-control and cohort studies. BMJ (Clin. Res. ed.).

[66] Oliver D., Papaioannou A., Giangregorio L., Thabane L., Reizgys K., Foster G. (2008). A systematic review and meta-analysis of studies using the STRATIFY tool for prediction of falls in hospital patients: How well does it work?. Age Ageing.

[67] Dalal N., Triggs B. Histograms of oriented gradients for human detection.

[68] The Geometry Center: Computing cOnstrained Delaunay Triangulation Algorithm. http://www.geom.uiuc.edu/~samuelp/del_project.html.

[69] Belbachir A., Litzenberger M., Schraml S., Hofstatter M., Bauer D., Schon P., Humenberger M., Sulzbachner C., Lunden T., Merne M. CARE: A dynamic stereo vision sensor system for fall detection.

[70] Kuris B., Dishongh T. (2006). SHIMMER-Sensing Health with Intelligence Modularity Mobility, and Experimental Reusability. Intel Corp..

[71] Wathen-Dunn W. (1967). Models for the Perception of Speech and Visual Form: Proceedings of a Symposium.

[72] Laptev I. (2005). On space-time interest points. Int. J. Comput. Vis..

[73] Collin C., Wade D.T., Davies S., Horne V. (1988). The Barthel ADL Index: A reliability study. Int. Disabil. Stud..

